# *DIAPH2* gene polymorphisms and laryngeal cancer risk in men

**DOI:** 10.1007/s00432-024-05820-4

**Published:** 2024-06-07

**Authors:** Edyta Cichocka, Sylwia Górczyńska-Kosiorz, Maciej Misiołek, Wojciech Ścierski, Anna Koniewska, Grażyna Stryjewska-Makuch, Janusz Gumprecht

**Affiliations:** Medical University of Silesia, Katowice, Poland

**Keywords:** *DIAPH2* gene polymorphisms, Laryngeal cancer risk, Genetic factor

## Abstract

**Background:**

The *DIAPH2* gene is one of the genes commonly associated with laryngeal squamous cell carcinoma (LSCC). In our study, we considered the four polymorphisms of this gene, i.e. rs5920828, rs4322175, rs12851931 and rs5921830 as potential genetic risk factors for LSCC.

**Methods:**

We determined the genotyping of the genetic variants of *DIAPH2* in 230 male patients with histologically confirmed LSCC compared to the European population. Demographic and environmental exposure data of each subject were examined. To conduct the genetic tests, extraction of total DNA was performed. We genotyped all four variants in each patient and determined their frequencies.

**Results:**

In the case of the rs12851931 polymorphism in the *DIAPH2* gene, a significant difference was observed in the distribution of the T stage depending on the polymorphism. Heterozygotes were more often associated with T2 stage, while homozygotes were more likely to have higher tumor stages. The rs12851931 homozygotes of *DIAPH2* were statistically significantly more prevalent in smokers. The results suggested that rs12851931 polymorphism in *DIAPH2* could increase the onset risk of LSCC.

**Conclusions:**

Our results provide further information on the role of the *DIAPH2* gene in the pathogenesis of LSCC.

**Supplementary Information:**

The online version contains supplementary material available at 10.1007/s00432-024-05820-4.

## Introduction

Laryngeal squamous cell carcinoma (LSCC) is one of the most common malignancies of the head and neck. It is a significant clinical problem because of its high incidence and the lack of effective treatment. Due to higher rates of tobacco and alcohol use among males, LSCC is more prevalent in men than in women (the male-to-female ratio ranging from 5:1 to 10:1) (Talamini et al. 2002; Zhang et al. 2021). The incidence of LSCC varies across different countries in Europe. The highest rates are reported in Central and Eastern European countries, while the lowest rates are observed in Northern European countries (Igissin et al. 2023; Wierzbicka et al. 2016).

The cause and etiopathogenesis of LSCC are still not fully understood. Epidemiological studies have indicated environmental factors as significant risk factors for developing LSCC. Among the crucial risk factors, the following should be mentioned: tobacco and alcohol use, human papillomavirus (HPV) infection and prolonged exposure to certain chemicals (asbestos fibers, wood dust, chemicals in the metalworking industry and exhaust fumes) (Kreimer et al. 2005).

The incidence of LSCC is strongly associated with age and gender. It typically occurs in male patients over the age of 40, with the peak incidence in the sixth and seventh decades of life. The prognosis for advanced-stage LSCC is poorer, with 5-year survival rates typically below 50%.

In the era of the growing importance of personalized cancer therapy, including LSCC, it is essential to search for genetic markers that increase the risk of disease development and use them for diagnosis, prognosis and personalized treatment strategies for LSCC patients.

Several genetic markers for LSCC have already been identified, including TP53 mutations (Szczepny et al. 2018), mutations in the Ras family genes (H-K-N-Ras) (Bos 1988), *EGFR* (*epidermal growth factor receptor*) gene amplification or overexpression (Politi et al. 2019), *Cyclin D1* (*CCND1*) gene amplification (Kyrodimos et al. 2020), *FGFR3* (fibroblast growth factor receptor) gene mutations (Manterola et al. 2018), loss of heterozygosity (LOH) (Grbesa et al. 2008), DNA (deoxyribonucleic acid) methylation, *SLC22A23* gene (Ekizoglu et al. 2018), *protein tyrosine phosphatase receptor Type D* (*PTPRD*) gene and the rs3765142 polymorphism in *PTPRD* (Szaumkessel et al. 2017; Śnit et al. 2021), *hypermethylated-in-cancer-1* (*HIC1*) gene (Ekizoglu et al. 2018) and the rs9901806 polymorphism in the *HIC1* gene (Śnit et al. 2021; Dubuissez et al. 2020). *Diaphanous-related formin 2* (*DIAPH2*) is a member of the diaphanous-related formin family, which encodes proteins involved in cytoskeletal organization and cellular processes, such as cell division, migration and adhesion. The *DIAPH2* gene is located on the long arm (q) of chromosome X at position 21, 23. Several studies have highlighted the involvement of *DIAPH2* gene abnormalities in LSCC (Śnit et al. 2021; Kostrzewska-Poczekaj et al. 2019). Some studies have identified specific mutations in the *DIAPH2* gene in LSCC patients. *DIAPH2* mutations were present in approximately 4% of LSCCs, which was mainly related to *DIAPH2* gene expression. High expression of *DIAPH2* was associated with advanced tumor stage, lymph node metastasis and poor overall survival in LSCC patients. Alterations in *DIAPH2* increase cell motility and may contribute to the metastatic potential of LSCC (Kostrzewska-Poczekaj et al. 2019). Upregulation of *DIAPH2* in LSCC may act as an oncogene by inhibiting apoptosis through the ART/p53/caspase-3 pathway (Yang et al. 2019). So far, one of the *DIAPH2* gene polymorphisms has been associated with LSCC.

In the previous observation (2021) from our clinical center (Zabrze, Poland), we confirmed for the first time that rs6620138 polymorphism in the *DIAPH2* gene was associated with the pathogenesis of LSCC. Significant differences in allele distribution of *DIAPH2* rs6620138 in the case and control groups were observed (Śnit et al. 2021).

The rs6620138 polymorphism in the *DIAPH2* gene has been implicated in the development and progression of LSCC (Śnit et al. 2021). The rs6620138 polymorphism affects *DIAPH2* expression levels. In particular, individuals with specific alleles of rs6620138, such as the TG or GG genotypes, have been found to have lower *DIAPH2* expression compared to individuals with the TT genotype. The decreased expression of *DIAPH2* is associated with an increased risk of LSCC. Alterations in *DIAPH2* expression due to the rs6620138 polymorphism could potentially impact the ability of cells to migrate and invade, contributing to the development and progression of LSCC. Studies have suggested that the rs6620138 polymorphism may influence the response to specific anticancer treatments (Śnit et al. 2021). For example, individuals with the GG genotype of rs6620138 had a better response to chemotherapy compared to those with the TT genotype. This association may be attributed to the influence of *DIAPH2* on actin dynamics, which can impact the sensitivity of cancer cells to chemotherapy agents (Śnit et al. 2021).

In this study, we considered the following four polymorphisms of the *DIAPH2* gene: rs5920828, rs4322175, rs12851931 and rs5921830 as potential genetic risk factors for LSCC. All of the above polymorphisms have been poorly investigated.

The *DIAPH2* rs5920828 is a specific variant of the *DIAPH2* gene, which encodes for diaphanous-related formin-2 protein. As regards the specific variant (rs5920828), its role in disease susceptibility and biology is not well-established. However, genetic variants within the *DIAPH2* gene have been associated with several human disorders. For example, mutations in *DIAPH2* have been found in individuals with microcephaly and cataract, suggesting its involvement in brain development and eye health (Kostrzewska-Poczekaj et al. 2019).

The *DIAPH2* rs4322175 encodes for diaphanous-related formin 2 protein, which is involved in various cellular processes, including cell division, cell migration and cytoskeleton organization. The rs4322175 variant is associated with differences in the expression or activity of the *DIAPH2* protein. Studies have shown that individuals carrying the alertative C allele of rs4322175 may have altered *DIAPH2* protein levels or activity compared to individuals with the reference allele (T allele). The role of *DIAPH2* rs4322175 in different biological processes and diseases is still under investigation. Some research suggests this variant could be associated with susceptibility to some disorders. For example, one study found that individuals with the C allele of rs4322175 showed an increased risk of developing Alzheimer’s disease compared to those with the T allele (Labat-de-Hoz and Alonso 2021).

The *DIAPH2* rs12851931 is a genetic variant of the *DIAPH2* gene. The rs12851931 variant is associated with an increased risk of developing various diseases and conditions. In some studies, this genetic variant was associated with an increased risk of schizophrenia, bipolar disorder, autism spectrum disorder (ASD), an increased risk of ovarian cancer, colorectal cancer and early-onset myocardial infarction (Philippe et al. 1993).

The *DIAPH2* rs5921830 is a genetic variant of the *DIAPH2* gene that has also been associated with various diseases and conditions. One study found that individuals with the CC genotype of rs5921830 had a higher risk of developing colorectal cancer. Another study found an association with the development of ASD in males. The CC genotype was more prevalent in individuals with ASD compared to controls. The *DIAPH2* rs5921830 has been suggested to alter the expression or function of the *DIAPH2* protein, leading to dysregulation of cellular processes and potentially increasing disease susceptibility (https://www.proteinatlas.org/ENSG00000147202-DIAPH2/pathology).

## Purpose

The aim of our study was to continue previous research and find a link between the four polymorphisms of the *DIAPH2* gene and the risk of LSCC development. We determined the genotyping of the genetic variants of *DIAPH2* in 230 male patients with histologically confirmed LSCC compared to the European population. Due to the predominance of men in the incidence of LSCC and the fact that the collected material was obtained from only several women, we decided to standardize the study group and analyze only male patients.

## Methods

### Study population

The study protocol was approved by the Bioethics Committee of the Medical University of Silesia (Poland). The study population consisted of 230 subjects with histologically confirmed LSCC treated in the Department of Otorhinolaryngology and Laryngological Oncology, Medical University of Silesia (Zabrze, Poland), Department of Laryngology and Laryngological Oncology, Upper Silesian Medical Centre of Silesian Medical University, (Katowice, Poland) and Department of Otolaryngology, Head and Neck Surgery, Holy-Cross Cancer Centre (Kielce, Poland). All patients underwent laryngectomy and had no distant metastases (M0 according to the TNM classification). Demographic and environmental exposure data of each subject were examined, including sex, age, smoking, alcohol consumption, a history of diseases and a family history of cancer. Information on smoking and alcohol consumption was obtained from the medical history. For smokers, the number of pack-years smoked was calculated. Non-smokers were defined as patients who had stopped smoking at least 10 years before cancer diagnosis. To assess alcohol consumption, patients declared the amount of alcohol consumed per week. Increased exposure to alcohol was considered when the alcohol intake was more than 280 mg of pure alcohol per week (approximately 13 0.5-L bottles of beer, 3 0.75-L bottles of wine and 0.8 L of vodka). To assess the genetic predisposition to cancer, we took into account the occurrence of cancer, particularly in the first- and second-degree relatives.

The study material was obtained in cooperation with the Department of Pathomorphology at the Medical University of Silesia (Zabrze, Poland).

For the European population, we used the most frequently selected database, i.e. NCBI. Unfortunately, there is no division into gender or age in the database, only the final size group, ethnicity and allele distribution for the selected SNPs. The European population used to compare the distribution of the polymorphisms (identifier: BioSample: SAMN10492695) was taken from the NCBI database on 5th January 2024. The largest population was selected for comparison.

- for rs12851931, the reference population consisted of 19,993 people; gender breakdown was not included in the data (https://www.ncbi.nlm.nih.gov/snp/rs12851931)
- for rs4322175, the reference population consisted of 219,852 people; gender breakdown was not included in the data (https://www.ncbi.nlm.nih.gov/snp/rs4322175; https://www.ncbi.nlm.nih.gov/snp/rs5920828)
- for rs5920828, the reference population consisted of 14,150 people; gender breakdown was not included in the data (https://www.ncbi.nlm.nih.gov/snp/rs5921830)
- for rs5921830, the reference population consisted also of 14,150 people; gender breakdown was not included in the data (https://support.rstudio.com/hc/en-us/articles/206212048-Citing-RStudio).

### Genetic analysis

To conduct the genetic tests, extraction of total DNA was performed with the MagCore Genomic DNA FFPE (formalin-fixed paraffin—embedded) One-Step kit (IVD) using MagCore®Compact. We used a NanoDrop spectrophotometer (Thermo Scientific, Waltham, USA) to measure the concentration and purification of the DNA. The genetic material was then used to genotype four variants of the *DIAPH2* gene. The SNPs identifiers were as follows: rs4322175, rs5920828, rs5921830 and rs12851931. We used TaqMan SNP genotyping assays (ThermoFisher, USA) to discriminate alleles (Table S1). For PCR, we used FastStart Essential DNA Probes Master (Roche, Switzerland) for real-time PCR using the LifeCycler 96 thermocycler (Roche, Switzerland). We genotyped all four variants in each patient and determined their frequencies.

### Statistical analysis

Data were presented as the number of cases with the percentage for qualitative variables. To assess the normality of the variables, we used a histogram and a quantile–quantile (QQ) plot. Quantitative variables with a normal distribution were presented as means with standard deviation and variables with deviations from the normal distribution were presented as the median with the first and third quartiles. For statistical analyses of quantitative variables with the normal distribution, the Student’s *t* test was used. The Wilcoxon test was applied for quantitative variables with the deviation from the normal distribution. The results for *smokers* and *drinkers* were presented in boxplots. The comparison of qualitative variables was based on the Pearson’s chi-squared test. The GLM method was used to assess the correlations between the polymorphisms and other variables. The comparison of allele frequencies in the study population with the general population was made using the test for proportions. The analysis was performed using the R language in the RStudio environment (Wickham et al. 2019) and Tidyverse and Janitor packages (Zhang et al. 2022). *p* values <0.05 were considered statistically significant.

## Results

A total of 230 patients with LSCC were included in the study group. The general characteristics of the study population are given in Table 1. The mean age of the study population was 63.7 years. 44.80% of patients were diagnosed with T4 cancer and 41.70% with N2 stage. Other stages are listed in Table 2. None of the patients had distant metastases (M0 stage = 100%; Table 1). Recurrence occurred in 28.30% of subjects regardless of the stage of the disease. Concomitant cancers were diagnosed in 8.20% of patients, the most common being glottis cancer (3.30%). The 5-year survival was found in 46.96%, while 53.04% of patients died. A family history of cancer was reported by 7.7% of patients (Table 1). The median pack-year history of smoking was 25 years. 84.30% of patients were smokers, and 79.10% were drinkers. 68.70% of patients were both smokers and drinkers (Table 2). As shown in Table 3, the mean age of smokers was 63.7 years, and 63.4 years in non-smokers (*p* = 0.83, NS). The mean age of drinkers was 63.2 years compared to 65.3 years for non-drinkers (*p* = 0.099, NS). The mean age of drinkers and smokers was 63.4 years compared to 64.2 years for both non-smokers and non-drinkers (*p* = 0.52, NS).

**Table 1:** The general characteristics of the study population and TNM classsification

	*n*	Percent (%)
Sex (male)	230	100.00
Five-year cancer survival	108	46.96
Death	122	53.04
Cancer recurrence	65	28.30
Smokers/non-smokers	194	84.30/15.7
Drinkers/non-drinkers	182	79.10/20.9
Smokers and drinkers	158	68.70
Other cancer history	19	8.26
Family history of cancer	18	7.80
TNM classification		
T1	4	1.90
T2	14	6.70
T3	85	40.50
T4	94	44.80
Tis	3	1.40
Tx	10	4.80
*N* (%)		
N0	79	38.30
N1	40	19.40
N2	86	41.70
N3	1	0.50
*M* = M0 (%)	230	100.00

**Table 2:** The age and pack-year history of smoking

Variable	*N*	Min	Max	Median	q1	q3	Mean	SD
Age^a^	230	42	92	64	58	69	63.7	8.49
Pack-year history of smoking	127	0	120	25	0	40	26.4	23.9

^a^Age—normal distribution

**Table 3:** The correlation between the mean age and drinkers, smokers and both drinkers and smokers

	Variable	*N*	Median	q1	q3	Mean	SD	*p* value
Smokers								
0	Age^a^	36	66	58	68.2	63.4	8.22	0.830
1		194	64	58	69	63.7	8.56	
Drinkers								
0	Age	48	65.5	60.8	70	65.3	7.48	0.099
1		182	64	57	68	63.2	8.7	
Smokers and drinkers								
0	Age	72	66	59	69	64.2	8.09	0.520
1		158	64	57	69	63.4	8.68	

“0”—no-smokers, no-drinkers, no-smokers and no-drinkers

“1”—smokers, drinkers, smokers and drinkers

^a^Age—normal distribution

Allele distribution in smokers, drinkers and both smokers and drinkers is given in Table 4. Homozygotes of rs12851931 polymorphism in *DIAPH2* were statistically significantly more prevalent in smokers than in non-smokers (Table 4). The distribution of particular alleles in the study population is given in Table 5. The allele distribution of rs5920828 and rs5921830 polymorphisms of *DIAPH2* differed statistically from the distribution ​​reported in the European population (Table 6). The rs4322175 and rs12851931 polymorphisms in *DIAPH2* showed a trend to statistical significance (*p* = 0.08 and *p* = 0.07, respectively). The assessment of the relationship between the polymorphisms and the TNM stages showed no difference in three polymorphisms (rs5920828, rs4322175 and rs5921830). In the case of the rs12851931 polymorphism in the *DIAPH2* gene, a significant difference was observed in the distribution of the T stage depending on the polymorphism. Heterozygotes were more often associated with T2 stage, while homozygotes were more likely to have higher tumor stages (Table 7). GLM analysis for the polymorphisms and cancer history, survival and cancer recurrence is given in Tables 8, 9, and 10. No statistically significant differences were found between cancer history, survival, risk of cancer recurrence and the analyzed polymorphisms.

**Table 4:** The allele distribution in smokers, drinkers and both smokers and drinkers

Smokers			
	0	1	*p*
	36	194	
DIAPH2_rs5920828 (%)			0.679
AA	15 (45.5)	60 (37.3)	
AT	2 (6.1)	11 (6.8)	
TT	16 (48.5)	90 (55.9)	
DIAPH2_rs4322175 (%)			0.503
CC	18 (52.9)	67 (43.2)	
CT	2 ( 5.9)	7 (4.5)	
TT	14 (41.2)	81 (52.3)	
DIAPH2_rs12851931 (%)			0.009
AA	13 (37.1)	65 (40.1)	
AG	7 (20.0)	8 (4.9)	
GG	15 (42.9)	89 (54.9)	
DIAPH2_rs5921830 (%)			0.718
CC	13 (37.1)	60 (37.0)	
CT	4 (11.4)	12 (7.4)	
TT	18 (51.4)	90 (55.6)	

Drinkers			
	0	1	*p*
*n*	48	182	
DIAPH2_rs5920828 (%)			0.755
AA	17 (37.8)	58 (38.9)	
AT	2 (4.4)	11 (7.4)	
TT	26 (57.8)	80 (53.7)	
DIAPH2_rs4322175 (%)			0.183
CC	20 (43.5)	65 (45.5)	
CT	0 (0.0)	9 (6.3)	
TT	26 (56.5)	69 (48.3)	
DIAPH2_rs12851931 (%)			0.563
AA	20 (44.4)	58 (38.2)	
AG	2 (4.4)	13 (8.6)	
GG	23 (51.1)	81 (53.3)	
DIAPH2_rs5921830 (%)			0.51
CC	19 (41.3)	54 (35.8)	
CT	2 (4.3)	14 (9.3)	
TT	25 (54.3)	83 (55.0)	

Both drinkers and smokers			
	0	1	*p*
*n*	72	158	
DIAPH2_rs5920828 (%)			0.648
AA	25 (37.9)	50 (39.1)	
AT	3 (4.5)	10 (7.8)	
TT	38 (57.6)	68 (53.1)	
DIAPH2_rs4322175 (%)			0.656
CC	32 (47.1)	53 (43.8)	
CT	2 (2.9)	7 (5.8)	
TT	34 (50.0)	61 (50.4)	
DIAPH2_rs12851931 (%)			0.496
AA	28 (41.2)	50 (38.8)	
AG	7 (10.3)	8 (6.2)	
GG	33 (48.5)	71 (55.0)	
DIAPH2_rs5921830 (%)			0.916
CC	25 (36.2)	48 (37.5)	
CT	5 (7.2)	11 (8.6)	
TT	39 (56.5)	69 (53.9)	

“0”—no-smokers, no-drinkers, no-smokers and no-drinkers

“1”—smokers, drinkers, smokers and drinkers

**Table 5:** The alleles of the polymorphisms in the study population

	*N*	Percent (%)
DIAPH2_rs5920828		
AA	75	38.70
AT	13	6.70
TT	106	54.60
DIAPH2_rs5920828_allel_A	88	45.40
DIAPH2_rs5920828_allel_T	119	61.30
DIAPH2_rs4322175		
CC	85	45.00
CT	9	4.80
TT	95	50.30
DIAPH2_rs4322175_allel_C	94	49.70
DIAPH2_rs4322175_allel_T	104	55.00
DIAPH2_rs12851931		
AA	78	39.60
AG	15	7.60
GG	104	52.80
DIAPH2_rs12851931_allel_A	93	47.20
DIAPH2_rs12851931_allel_G	119	60.40
DIAPH2_rs5921830		
CC	73	37.10
CT	16	8.10
TT	108	54.80
DIAPH2_rs5921830_allel_C	89	45.20
DIAPH2_rs5921830_allel_T	124	62.90

**Table 6:** The allele distribution in the study population versus the European population

	Frequency in the general population (European)	Frequency in the study population	*n*	%	Sample estimates	*p* value	95% CI	
DIAPH2 rs5920828								
A	0.616	0.454	88	45.40	0.383	0.00	0.390	0.518
T	0.384	0.613	119	61.30	0.517		0.550	0.676
DIAPH2 rs4322175								
C	0.449	0.497	94	49.70	0.409	0.08	0.432	0.562
T	0.551	0.550	104	55.00	0.452		0.486	0.614
DIAPH2 rs12851931								
A	0.534	0.472	93	47.20	0.404	0.07	0.407	0.537
G	0.466	0.604	119	60.40	0.517		0.541	0.667
DIAPH2 rs5921830								
C	0.567	0.452	89	45.20	0.387	0.00	0.388	0.516
T	0.433	0.629	124	62.90	0.539		0.567	0.691

**Table 7:** The relationship between the polymorphisms and the TNM stages (Pearson’s chi-squared test)

Stratified by DIAPH2_rs5920828				
	AA	AT	TT	*p* test
*n*	75	13	106	
Stage_T (%)				0.666
T1	1 (1.5)	0 (0.0)	2 (2.1)	
T2	4 (6.1)	0 (0.0)	6 (6.2)	
T3	28 (42.4)	3 (27.3)	40 (41.2)	
T4	30 (45.5)	7 (63.6)	42 (43.3)	
Tis	0 (0.0)	1 (9.1)	2 (2.1)	
Tx	3 (4.5)	0 (0.0)	5 (5.2)	
Stage_N (%)				0.677
N0	23 (35.4)	4 (40.0)	41 (42.7)	
N1	10 (15.4)	3 (30.0)	18 (18.8)	
N2	32 (49.2)	3 (30.0)	36 (37.5)	
N3	0 (0.0)	0 (0.0)	1 (1.0)	

Stratified by DIAPH2_rs4322175				
	CC	CT	TT	*p* test
*n*	85	9	95	
Stage_T (%)				0.514
T1	2 (2.6)	0 (0.0)	1 (1.2)	
T2	6 (7.7)	1 (11.1)	4 (4.9)	
T3	32 (41.0)	3 (33.3)	36 (43.9)	
T4	33 (42.3)	4 (44.4)	35 (42.7)	
Tis	2 (2.6)	1 (11.1)	0 (0.0)	
Tx	3 (3.8)	0 (0.0)	6 (7.3)	
Stage_N (%)				0.821
N0	36 (47.4)	3 (37.5)	31 (37.8)	
N1	13 (17.1)	2 (25.0)	14 (17.1)	
N2	27 (35.5)	3 (37.5)	36 (43.9)	
N3	0 (0.0)	0 (0.0)	1 (1.2)	

Stratified by DIAPH2_rs5921830				
	CC	CT	TT	*p* test
*n*	73	16	108	
Stage_T (%)				0.186
T1	2 (3.1)	0 (0.0)	1 (1.0)	
T2	4 (6.2)	2 (14.3)	6 (6.1)	
T3	19 (29.7)	5 (35.7)	49 (49.5)	
T4	35 (54.7)	6 (42.9)	37 (37.4)	
Tis	0 (0.0)	1 (7.1)	2 (2.0)	
Tx	4 (6.2)	0 (0.0)	4 (4.0)	
Stage_N (%)				0.925
N0	23 (36.5)	6 (46.2)	41 (41.8)	
N1	12 (19.0)	3 (23.1)	17 (17.3)	
N2	28 (44.4)	4 (30.8)	39 (39.8)	
N3	0 (0.0)	0 (0.0)	1 (1.0)	

Stratified by DIAPH2_rs12851931				
	AA	AG	GG	*p* test
*N*	78	15	104	
Stage_T (%)				0.025
T1	3 (4.2)	0 (0.0)	0 (0.0)	
T2	4 (5.6)	4 (26.7)	3 (3.3)	
T3	27 (38.0)	5 (33.3)	42 (46.2)	
T4	32 (45.1)	5 (33.3)	41 (45.1)	
Tis	1 (1.4)	1 (6.7)	1 (1.1)	
Tx	4 (5.6)	0 (0.0)	4 (4.4)	
Stage_N (%)				0.729
N0	31 (44.3)	5 (35.7)	33 (36.7)	
N1	9 (12.9)	3 (21.4)	20 (22.2)	
N2	30 (42.9)	6 (42.9)	36 (40.0)	
N3	0 (0.0)	0 (0.0)	1 (1.1)

**Table 8:** GLM analysis for the polymorphisms and cancer history

DIAPH2 rs5920828	Cases (*n* = 89)		Controls (*n* = 9)		*p* value	OR (95% CI)
Genotypes	*N*	%	*n*	%		
AA	34	38.2	4	44.4	–	1.0 (Reference)
AT	7	7.9	0	0	0.992	2E−07 (–)
TT	48	53.9	5	55.6	0.863	0.885 (0.22–3.80)
AA+AT	41	46.1	4	44.4	0.926	0.937 (0.22–3.76)

DIAPH2 rs4322175	Cases (*n* = 83)		Controls (*n* = 9)		*p* value	OR (95% CI)
Genotypes	*N*	%	*n*	%		
CC	41	49.4	3	33.3	–	1.0 (Reference)
CT	3	3.6	0	0	0.995	3.21E−07 (–)
TT	39	47	6	66.7	0.316	2.1 (0.52–10.50)
CC+CT	44	53	3	33.3	0.272	0.443 (0.09–1.80)

DIAPH2 rs12851931	Cases (*n* = 91)		Controls (*n* = 9)		*p* value	OR (95% CI)
Genotypes	*N*	%	*n*	%		
AA	34	37.4	3	33.3	–	1.0 (Reference)
AG	13	14.3	0	0	0.993	9.8E−08 (–)
GG	44	48.4	6	66.7	0.558	1.55 (0.38–7.74)
AA+AG	47	51.6	3	33.3	0.303	0.468 (0.09–1.89)

DIAPH2 rs5921830	Cases (*n* = 91)		Controls (*n* = 9)		*p* value	OR (95% CI)
Genotypes	*N*	%	*n*	%		
CC	34	37.4	2	22.2	–	1.0 (Reference)
CT	8	8.8	0	0	0.995	1.47E−07 (–)
TT	49	53.8	7	77.8	0.286	2.43 (0.55–17.00)
CC+CT	42	46.2	2	22.2	0.185	0.333 (0.05–1.47)

**Table 9:** GLM analysis for the polymorphisms and survival

DIAPH2 rs5920828	Cases (*n* = 74)		Controls (*n* = 55)		*p* value	OR (95% CI)
Genotypes	*N*	%	*n*	%		
AA	27	36.5	26	47.3	–	1.0 (Reference)
AT	4	5.4	4	7.3	0.960	1.04 (0.22–4.81)
TT	43	58.1	25	45.5	0.176	0.604 (0.29–1.25)
AA+AT	31	41.9	30	54.5	0.156	1.66 (0.83–3.39)

DIAPH2 rs4322175	Cases (*n* = 72)		Controls (*n* = 52)		*p* value	OR (95% CI)
Genotypes	*N*	%	*n*	%		
CC	32	44.4	25	48.1	–	1.0 (Reference)
CT	2	2.8	3	5.8	0.493	1.92 (0.30–15.4)
TT	38	52.8	24	46.2	0.569	0.808 (0.39–1.68)
CC+CT	34	47.2	28	53.8	0.467	1.3 (0.64–2.68)

DIAPH2 rs12851931	Cases (*n* = 74)		Controls (*n* = 57)		*p* value	OR (95% CI)
Genotypes	*N*	%	*n*	%		
AA	30	40.5	18	31.6	–	1.0 (Reference)
AG	6	8.1	8	14	0.196	2.22 (0.67–7.77)
GG	38	51.4	31	54.4	0.424	1.36 (0.64–2.91)
AA+AG	36	48.6	26	45.6	0.730	0.885 (0.44–1.77)

DIAPH2 rs5921830	Cases (*n* = 74)		Controls (*n* = 57)		*p* value	OR (95% CI)
Genotypes	*N*	%	*n*	%		
CC	30	40.5	24	42.1	–	1.0 (Reference)
CT	5	6.8	4	7	1.000	1 (0.23–4.18)
TT	39	52.7	29	50.9	0.842	0.929 (0.45–1.92)
CC+CT	35	47.3	28	49.1	0.836	1.08 (0.54–2.15)

**Table 10:** GLM analysis for the polymorphisms and cancer recurrence

DIAPH2 rs5920828	Cases (*n* = 135)		Controls (*n* = 57)		*p* value	OR (95% CI)
Genotypes	*N*	%	*N*	%		
AA	50	37	24	42.1	–	1.0 (Reference)
AT	11	8.1	2	3.5	0.229	0.379
TT	74	54.8	31	54.4	0.678	0.873
AA+AT	61	45.2	26	45.6	0.957	1.02 (0.54–1.89)

DIAPH2 rs4322175	Cases (*n* = 132)		Controls (*n* = 55)		*p* value	OR (95%CI)
Genotypes	*N*	%	*N*	%		
CC	61	46.2	23	41.8	–	1.0 (Reference)
CT	6	4.5	3	5.5	0.706	1.33
TT	65	49.2	29	52.7	0.611	1.18
CC+CT	67	50.8	26	47.3	0.664	0.87 (0.46–1.63)

DIAPH2 rs12851931	Cases (*n* = 137)		Controls (*n* = 58)		*p* value	OR (95% CI)
Genotypes	*N*	%	*N*	%		
AA	52	38	25	43.1	–	1.0 (Reference)
AG	12	8.8	3	5.2	0.343	0.52
GG	73	53.3	30	51.7	0.630	0.855
AA+AG	64	46.7	28	48.3	0.842	1.06 (0.57–1.97)

DIAPH2 rs5921830	Cases (*n* = 138)		Controls (*n* = 57)		*p* value	OR (95% CI)
Genotypes	*N*	%	*N*	%		
CC	49	35.5	23	40.4	–	1.0 (Reference)
CT	11	8	4	7	0.688	0.775
TT	78	56.5	30	52.6	0.548	0.819
CC+CT	60	43.5	27	47.4	0.619	1.17 (0.63–2.18)

## Discussion

Laryngeal squamous cell carcinoma (LSCC) is one of the most common malignant neoplasms of the respiratory system (Talamini et al. 2002). It is more prevalent in males and often occurs in individuals over the age of 40. Genetic factors also play an essential role in the etiopathogenesis of LSCC. Genetic research on LSCC explores mutations, copy number variations and epigenetic changes. The *DIAPH2* gene is one of the potential genetic factors of LSCC.

The polymorphisms we have analyzed are still poorly understood. There are only a few studies on their relationship with cancers or other diseases.

We examined the distribution of the studied genotypes and alleles in the whole group of subjects and compared the results to the distribution in the European population. The allele distribution of two polymorphisms (rs5920828 and rs5921830) differed statistically from the distribution found in the European population. The next two polymorphisms (rs4322175 and rs12851931) showed a trend to statistical significance and presumably the difference would be significant in the case of a larger group size. We are aware that trend is not a significant value in statistics but used this statement to highlight the proximity of the *p* value to 0.05. In our opinion, the described polymorphisms may have the clinical relevance and hence further studies are warranted.

Further analysis showed that three of the studied polymorphisms (rs5920828, rs4322175 and rs5921830) showed no significant difference in relation to the TNM stages. However, a significant difference was observed for rs12851931 in the distribution of the T stage depending on the polymorphism. Heterozygotes of rs12851931 variant of the *DIAPH2* gene were more often associated with T2 stage, while homozygotes were more likely to have higher tumor stages. The total number of patients with stage T2 was 11. Therefore, the association of heterozygote of rs12851931 variant of the *DIAPH2* should be interpreted with caution, which is a limitation of the study. However, despite the small number of patients, their number was in fact sufficient to obtain the statistical significance. Even more in the case of the other polymorphisms, the total number of patients with T2 was similar (from 10 to 12 patients) and we found no correlation.

As mentioned in Introduction of our manuscript and considering the fact that *DIAPH2* gene mutations were present in approximately a few percent of LSCCs and that high expression of *DIAPH2* was associated with advanced tumor stage, lymph node metastasis and poor overall survival, it should be stressed that our results may have the practical aspect and may allow the creation, after confirmation in other studies, of an early marker of LSCC which can be useful in the diagnosis of LSCC at an early stage in some risk groups, such as smokers.

The importance of our research (even though it was conducted on such a small group of patients) also increases knowledge related to this very important area in the field of oncology and may be the basis for practical implementation in the future.

In the study population, most patients were homozygous (AA-39.6%, GG-52.8%), which may indicate the relationship between rs12851931 and an increased risk of more advanced LSCC. Furthermore, homozygotes of rs12851931 polymorphism of *DIAPH2* were statistically significantly more prevalent in smokers. The rs12851931 variant of the *DIAPH2* gene was associated with a higher risk of cancer development (ovarian cancer or colorectal cancer) and could be related to LSCC, as indicated in our study (Zhang et al. 2022; Lin et al. 2014).

It can be suggested that people with the above polymorphism could have a higher risk of developing LSCC than those without them.

However, if our findings are confirmed by other authors, the determination of the rs12851931 variant of the *DIAPH2* gene or other variants of the *DIAPH2* gene that we have analyzed may have practical significance in predicting the risk of developing LSCC.

Importantly, our study and two previous Polish studies (Kostrzewska-Poczekaj et al. 2019; Śnit et al. 2021) have indicated an association of the *DIAPH2* gene variants with the risk of LSCC.

The study provides insights into the genetic variants of the *DIAPH2* gene in relation to LSCC. The findings emphasize the complexity of LSCC etiology and suggest that additional factors may contribute to our clinical outcomes apart from the specific *DIAPH2* gene polymorphisms.

Further research is warranted to fully understand the role of *DIAPH2* and other genetic markers in LSCC. Studying the functions and potential therapeutic implications of these markers may contribute to the development of targeted therapies for LSCC in the future.

The limitation of our study was a relatively small sample size and only male population.

## Conclusions

Our study showed that the rs12851931 polymorphism in the *DIAPH2* gene could be associated with the pathogenesis of LSCC in male. Further studies should be conducted on larger study groups, including other races and the female population to confirm the above findings. Our research (even though it was conducted on such a small group of patients) also increases knowledge related to this very important area in the field of oncology and may be the basis for practical implementation in the future.

### Supplementary Information

Below is the link to the electronic supplementary material.

Supplementary file1 (DOCX 17 KB)

## Data Availability

No datasets were generated or analysed during the current study.
