# Supplementary material for: DIAPH2 gene polymorphisms and laryngeal cancer risk in men
Source: J Cancer Res Clin Oncol. 2024 Jun 7;150(6):295. doi: 10.1007/s00432-024-05820-4 (PMC11156712; doi:10.1007/s00432-024-05820-4)
Supplement: Supplementary file 1 — Supplementary file1 (DOCX 17 KB) [file 432_2024_5820_MOESM1_ESM.docx]

Table S1. DIAPH2 variants information.

| **SNP** | **Ref** | **Alt** | **Position** | **HGVS** | **Functional Consequence** | **Context Sequence [VIC/FAM]** |
| --- | --- | --- | --- | --- | --- | --- |
| rs4322175 | C | T | chrX:97148507 (GRCh38.p14) | NC_000023.11:g.97148507C>T | Intron variant | TTTAATTATTTGCTTCTATCTGTAC**[C/T]**TCCTCTTGAGTTTCTTAGTAACAAA |
| rs5920828 | A | T | chrX:97194964 (GRCh38.p14) | NC_000023.11:g.97194964A>T | Intron variant | GGGAGTATGGTCCCAACCTTACCAT**[A/T]**TTCTAAAATAAATTTTGTAATGATT |
| rs5921830 | T | C | chrX:97464822 (GRCh38.p14) | NC_000023.11:g.97464822T>C | Intron variant | GATCATCATTTTGGCTACATAAATA**[T/C]**ATATATATGTATGTTTTTGTTGTTG |
| rs12851931 | A | G | chrX:97138548 (GRCh38.p14) | NC_000023.11:g.97138548A>T | Intron variant | CAGATGATTAAATTAAAATTGCTGT**[A/G]**TATGGTTTATTAACTGATGATTGCA |

SNP - single nucleotide variations

Ref - referentive allele

Alt - alternative allele

Position - position in the genome according to Homo sapiens (human) genome assembly GRCh38 (hg38_HC) GRCh38.p14

HGVS- The Human Genome Variation Society (HGVS) nomenclature standard

Functional Consequence- SNP position and function

Context Sequence [VIC/FAM]- context sequence TaqMan SNP genotyping assays
